# A Narrative Review on the Usage of Surgical Skin Grafting, Acitretin, and Tacrolimus in the Treatment of Hailey-Hailey Disease

**DOI:** 10.7759/cureus.19704

**Published:** 2021-11-18

**Authors:** Shreya Reddy, Hetal Brahmbhatt

**Affiliations:** 1 Biology, Creighton University, Omaha, USA; 2 Psychiatry, Mercy General Hospital, Sacramento, USA

**Keywords:** lesions, surgical skin grafting, acitretin, tacrolimus, hailey-hailey

## Abstract

Hailey-Hailey disease is a rare genetic disease that causes irregular blistering. The irregular blistering is also usually accompanied by skin lesions in the affected skin area. The symptoms and signs of Hailey-Hailey disease differ from one case to another. There is no one standard treatment method for Hailey-Hailey disease. However, there are certain treatment methods that do show some promise. This review will analyze the use and fruitfulness of surgical skin grafting, tacrolimus, and acitretin in multiple settings to treat Hailey-Hailey disease. Surgical skin grafting is done by removing the epidermis and a portion of the dermis, if not all of the dermis, healthy skin from a different part of the body, and transplanting it to the damaged area of the body. Acitretin is a retinoid that is a derivative of vitamin A that reduces abnormal differentiation of keratinocytes and inflammation which prove useful for helping skin diseases. Tacrolimus is an immunosuppressive drug that works by limiting the activity of the immune system to prevent it from producing substances that contribute to the redness and dryness of the skin, making it a candidate to be used for Hailey-Hailey disease treatment. The understood results of tacrolimus, acitretin, and surgical skin grafting on Hailey-Hailey disease are very limited and should be given more attention by healthcare leaders to the potential outcomes of these treatments on patients who have Hailey-Hailey disease.

## Introduction and background

Hailey-Hailey disease, also known as benign familial pemphigus, is a rare skin condition that affects around 1/50,000 of the general population. This condition affects both males and females equally [1]. The disease usually becomes most noticeable during puberty, but symptoms can appear at any time. The symptoms of Hailey-Hailey can vary but usually consist of skin erosions, skin vesicles, hyperkeratosis, and erythema [2]. Hailey-Hailey disease is inherited in an autosomal dominant way, but some cases are due to a mutated gene, ATP2C1, and therefore, can occur with patients who do not have a family history of the condition. 

Hailey-Hailey appears as painful rashes, lesions, blistering, and varying degrees of redness. The disease most commonly occurs in the areas where skin folds, including but not limited to the neck, axilla, and groin. Flaccid vesicles or bullae are the primary lesions, but they are hardly noticeable as they easily rupture and leave macerated erosions, sometimes with crusting [3]. There is a mutation in the ATP2C1 gene that codes for chromosome 3q21 that encodes adenosine triphosphate (ATP)-powered calcium pump protein hSPCA1 of epidermal cells, resulting in abnormal keratinocyte adhesion which is linked to Hailey-Hailey [3].

There is no one regular procedure to treat the Hailey-Hailey disease, but there are methods that have been experimented with. Patients with Hailey-Hailey are instructed to avoid conditions such as sunburn, sweating, friction, and to keep affected areas dry [1]. Additionally, cool compresses, dressings, mild corticosteroid creams, and topical antibiotics have shown they can be effective in treating mild cases. However, there is a lack of knowledge of treatment options for those who have moderate to severe Hailey-Hailey. This review will examine surgical skin grafting, tacrolimus, acitretin, and their respective effects on the Hailey-Hailey disease.

This research paper aims to explore the effects of surgical skin grafting, tacrolimus, and acitretin on the Hailey-Hailey disease. A literature search of articles published between 1970 and 2021 using PubMed was performed using the keywords “Hailey-Hailey,” “tacrolimus,” “surgical skin grafting,” “acitretin,” and “lesions.” Cases that included infants, elderly, allergies related to acids and steroids, existing history of skin surgeries, and skin trauma were excluded. Titles and abstracts were analyzed to ensure patients were not in a compromised state prior to the treatment of Hailey-Hailey. The articles reviewed included case studies and group studies, but most studies were case studies, most likely due to the rareness of Hailey-Hailey disease. The articles were analyzed for what type of treatment they used for the Hailey-Hailey disease, the concentration of the treatment method or where the surgery was done, and the overall results on the skin affected by the Hailey-Hailey disease. Results from the studies done were analyzed in a mixed-methodology manner, using both quantitative and qualitative analysis to create a holistic review. The quantitative analysis included percentages of effectiveness in some of the treatments, such as how many patients had improvements with elements of their condition, such as swelling, redness, and intensity of the blisters. The qualitative analysis included descriptions of visual elements of the Hailey-Hailey disease, such as the examination of the appearance of the lesions and redness of the skin. Forty-two articles were identified, and 33 of them were actually reviewed, due to the aforementioned criteria. Only articles that were available online and in English were included in this review.

## Review

Surgical skin grafting

Surgical skin grafting is a popular technique that is used for many skin conditions. Surgical skin grafting is a procedure that involves removing skin from one area of the body and transplanting it to a different area of the body, usually where the skin is damaged or lost [4]. In most cases, the surgery uses healthy skin from a person’s own body. Most skin grafts are done with the use of general anesthesia so the patient does not feel pain. When choosing which healthy skin to transplant, skin that matches the tone and texture of areas surrounding the graft site, is usually optimal. This takes the two top layers of skin from the donor site (the epidermis) and the layer under the epidermis (the dermis). This makes it a possible treatment for Hailey-Hailey because taking out some layers of the skin can help mitigate the effects of the disease, such as erosion. The donor site can be any area of the body that has healthy skin, which usually tends to be the back, upper arm, forearm, or abdomen [5]. The graft is carefully spread on the bare area where it is being transplanted. It is held in place either by gentle pressure from a well-padded dressing that covers it or by staples or a few small stitches [5].

Tacrolimus

Tacrolimus is a drug belonging to a class of immunosuppressants [6]. Tacrolimus is commonly used as an ointment to treat symptoms of eczema. Eczema is characterized by red, irritated, and itchy skin, which also occurs in patients with Hailey-Hailey disease, making the drug a promising treatment method. Tacrolimus works by weakening the immune system and relieving eczema. Tacrolimus does this by inhibiting calcineurin, which is involved in the production of interleukin-2, a molecule that promotes the development and proliferation of T cells as part of the body's adaptive immune response [7]. Tacrolimus ointment suppresses the immune system and the subsequent inflammation by inhibiting an enzyme (calcineurin) crucial for the multiplication of T-cells, cells that are required for activation of the immune system. In the treatment of Hailey-Hailey, the tacrolimus is usually used in its topical form and is applied to the damaged skin either once or twice a day.

Acitretin

Acitretin is a type of retinoid that is used to treat psoriasis and a variety of other severe skin disorders. Psoriasis is a skin condition that causes cells in the outer layer of the skin to grow faster than normal and build upon the surface of the skin. This leads to inflammation and redness, thickened areas of skin, much like Hailey-Hailey disease. Acitretin is a vitamin A derivative that works by allowing normal growth and development of the skin [8]. Vitamin A is useful to multiple layers of the skin, as it interrupts the process that breaks down collagen. Since it is an antioxidant, it increases the skin’s protection [9]. Acitretin works by binding to receptors in the body. Acitretin typically binds to retinoic acid receptors, located at the N-terminal of the body [9]. These receptors help normalize the speed of skin cell growth, reducing the effects of conditions that include psoriasis and other skin disorders like Hailey-Hailey that cause red and itchy skin [8]. Acitretin is usually administered in a topical form in Hailey-Hailey treatments, acitretin will continue to work after the patient stops taking it, but if the skin condition resurfaces, the patient may need to start taking it again [8].

Sites of the importance of surgical skin grafting

There are two sites of importance with respect to surgical skin grafting. The first is the site where the healthy skin is taken from. This specific site is known as the donor site. In the studies compiled under this review, it is not specified where exactly on their body each specific case had the healthy skin taken from. However, typical sites that are used in skin grafts for healthy skin are from the belly, back, bottom, or thighs [10]. The transplantation site is where the healthy skin is placed and is usually over the area where the epidermis (and possibly a portion of the dermis) was surgically removed. The groin and axilla are some of the most common areas of transplant [11-13]. Other areas tend to include the neck or the upper chest.

Dosage of tacrolimus and acitretin

The dosage of topical treatments is very important for skincare. The usual dosage for tacrolimus is usually 0.1% applied to the affected area twice a day [14-17]. There is a fine balance between too little tacrolimus and too much tacrolimus. Using too much tacrolimus will increase the severity of side effects. These side effects can include swollen and infected hair follicles, muscle pain, nausea, and oozing of blisters [18]. Additionally, an extreme overdose of tacrolimus can result in tremors, electrolyte disturbances, and elevated liver enzyme levels (which can cause abdominal pain. dark urine, and fatigue) [18]. However, too little of the tacrolimus can cause rejection of the transplanted skin. Other doses of tacrolimus that were experimented with are 0.03% and 0.1% and administered once a day [18-21]. The effects of acitretin are also reliant on its dosage. Too much acitretin can cause dry eyes, crusting of eyelids, peeling skin, taste change, and hair loss. In the most severe cases, excessive acitretin can also cause issues with the liver, specifically with inflammation. Acitretin can also decrease vision in the dark, resulting in a condition called night blindness that can occur spontaneously [8]. To treat Hailey-Hailey disease, acitretin tends to be administered at 25 mg per day [3,22-26]. However, in some of the studies incorporated in this review, acitretin is administered at different doses, usually ranging between 10 and 50 mg per day [25,27-29]. In some of the literature compiled in this review, acitretin can be administered alongside a different compound to try to maximize the improvement of the damaged skin [3,25,27].

Results of surgical skin grafting

The results from the surgical skin grafting procedures done by the experiments encompassed in this review tend to be positive. In one case study, surgical skin grafting was performed on the neck, axilla, and groin of the patient. After the first surgery, developed a recurrence of basal cell epithelioma at the edge of the skin graft of the left axilla, but the other sites seemed to be in remission. After a second skin graft of the whole left axilla, there was no further recurrence at the site of grafts. However, after one year, exacerbation of the condition occurred in the groin through the form of lesions [11]. In a group study, eight surgical skin grafts were done on patients in the groin, perineum, scrotum, and axilla. Only six patients were diagnosed with Hailey-Hailey disease, the other two patients were surgically operated on as controls. The six patients who were grafted remained free of disease in the regions treated [13]. According to Table 1, in another case study, the patient had a surgical skin graft done on the groin. After surgery, there was a noted absence of hair follicles, eccrine, and apocrine glands. However, the graft site remained relatively disease-free [12]. Overall, the results collected from the surgical skin grafting procedures seem to indicate that this procedure is effective for treating Hailey-Hailey disease.

**Table 1:** Summary of previous research on utilization of surgical skin grafting, acitretin, and tacrolimus to understand and treat Hailey-Hailey disease N/A: not applicable Many studies in this table are case studies, with two exceptions (Menz et al. [13] and Boehmer et al. [23]).

Author	Sample size	Type of treatment used	Dosage or where surgery was done	Results
Bitar and Giroux [11]	1	Surgical skin grafting	Neck, axilla, groin	After the first surgery, developed a recurrence of basal cell epithelioma at the edge of the skin graft. After a skin graft of the whole left axilla, no recurrence at the site of grafts, but an exacerbation of the condition occurred in groin lesions
Don et al. [12]	1	Surgical skin grafting	Groin	After surgery, absence of hair follicles, eccrine, and apocrine glands, graft site remained relatively disease-free
Menz et al. [13]	8	Surgical skin grafting	Groin, perineum, scrotum, axilla	The six patients who were grafted remained free of disease in the region treated. The other two were grafted as controls.
Laffitte et al. [14]	1	Tacrolimus	0.1% twice per day	Burning sensations, leading to progressive worsening of the lesions.
Rabeni and Cunningham [15]	1	Tacrolimus	0.1% twice per day	After six weeks, 50% decrease in plaques. After four months, only a 2 cm patch of erythema without erosions on the perineum.
Rubegni et al. [16]	1	Tacrolimus	0.1% twice per day	After two weeks, there was a dramatic improvement. After eight weeks, there was a complete remission.
Wang et al. [17]	1	Tacrolimus	0.1% twice per day	Lesions resolved within two months; no relapse was reported at one year to follow up.
Kaur and Sandhu [19]	1	Tacrolimus	0.03%	Responded well, lesions healed after around 10 weeks, no relapse observed at follow up a year later.
Sand and Thomsen [20]	1	Tacrolimus	0.1% per day	After one month, clearance of cutaneous lesions was observed.
Von Felbert et al. [21]	1	Tacrolimus	0.1% per day	Improved the lesions, but scar-like lesion on left labia minora developed.
Berger et al. [22]	1	Acitretin	25 mg per day	Improved dramatically after six months.
Boehmer et al. [23]	6	Acitretin	25 mg per day	Cleared skin of one patient, but worsened conditions for two others.
Dajani and Mutasim [24]	1	Acitretin	25 mg per day	Moderate improvement, but occurred rapidly.
Lapa and Breslavets [3]	1	Acitretin	25 mg per day, with narrowband phototherapy three times per week in flanks, axilla, under breasts, and mid and lower back	After two months, the patient had significant improvement and almost complete clearance of affected skin in all areas, maintained this at five months follow up.
Varada et al. [25]	1	Acitretin	Moved from 50 mg to 25 mg per day, with cyclosporine at 3.8 mg /kilograms of body weight per day	Four weeks after, lesions showed more significant improvement than ever, those in the axilla and groin disappeared completely, these results were sustained at six months follow up.
Vasudevan et al. [26]	1	Acitretin	25 mg per day	The patient started showing signs of improvement after four weeks, achieved complete regression after three months.
Usmani and Wilson [27]	1	Acitretin	20-30 mg per day, 10 mg in combination with cyclosporine of 1.2 mg /kilograms of body weight per day	The 20-30 mg dosage caused the rash to become more painful, erythematous, and macerated. The 10 mg in combination with cyclosporine dosage caused improvement in two days, eight months later the condition is relatively inactive.
Lipoff et al. [28]	1	Acitretin	10 mg per day, increased to 30 mg per day	10 mg caused the occasional occurrence of keratotic activity; the 30-mg dose eradicated any occurrence.
Naidoo et al. [29]	1	Acitretin	10 mg per day	Eradication of groin macerations and labial erosions
Tchernev et al. [30]	1	Tacrolimus	Concentration: N/A Dosage: given twice per day	Significant and fast improvement of skin lesions without itching or burning sensations
Mayuzumi et al. [31]	N/A	Tacrolimus	N/A	Induced suppression of ATP2C1 mRNA was inhibited by tacrolimus, improving skin appearance
Pagliarello et al. [32]	1	Tacrolimus	0.1% with 50% ZnOP per day	Improved the axilla, only darkened red around the edges of the axilla
Hurd et al. [33]	1	Tacrolimus	N/A	Treatment failed to improve skin at all
Duschet et al. [34]	1	Acitretin	N/A	Some improvement, but spontaneous resolution cannot be excluded.
Garayar et al. [35]	1	Acitretin	N/A	Treatment failed to improve skin at all.
Leblanc Jr et al. [36]	1	Acitretin	N/A	No success
Campbell et al. [37]	1	Acitretin	N/A	No lasting impact

Results of tacrolimus

The results of tacrolimus treatment also showed some potential. For instance, a case study in which 0.1% tacrolimus was applied to the area of skin where Hailey-Hailey disease was present twice a day, improved the skin. The patient’s lesions resolved within two months, and there no relapse was present at the one-year follow-up examination [17]. This is further supported by analysis of another case study in which the patient was given 0.1% tacrolimus twice a day. After two weeks, there was a dramatic improvement of the skin lesions and decreasing reddening of the skin. After eight weeks, there was a complete remission of the skin [16]. An additional patient was also given 0.1% tacrolimus twice a day. After six weeks, there was a 50% decrease in skin plaques, along with reduced reddening on the skin. After four months, there was only a 2 cm patch of erythema without erosions on the perineum [15]. However, in one case study, a patient was given 0.1% tacrolimus twice a day which led the patient to feel burning sensations, leading to progressive worsening of the lesions [14]. Another patient was given tacrolimus twice a day and experienced significant and fast improvement of skin lesions without itching or burning sensations [30]. Tacrolimus was administered in a different study at 0.03% daily, and the patient responded well. Their lesions healed after around 10 weeks, no relapse was observed at the follow-up a year later [19]. The patient in a different case study was given 0.1% tacrolimus once a day. After one month, complete clearance of cutaneous lesions was observed [20]. The same dosage of tacrolimus given once a day in another case study improved the patient’s lesions, but small, scar-like lesions on the left labia minora developed after two days of the operation [21]. In a different study, tacrolimus was found to induce suppression of the ATP2C1 gene. The mRNA was inhibited by tacrolimus, which was found to improve skin appearance by decreasing inflammation and redness [31]. As Table 1 shows, tacrolimus can also be used alongside different compounds to improve the skin. In one experiment, 0.1% tacrolimus was given to the patient with 50% ZnOP per day. This treatment improved the axilla, leaving only darkened red around the edges of the axilla [32]. Although, in one case study, a patient received tacrolimus as a treatment method for Hailey-Hailey, and no improvement was shown [33]. Tacrolimus procedures produced variable results, but nonetheless could be a promising method for treating Hailey-Hailey disease.

Results of acitretin

The treatment method of acitretin also produced fluctuating results in patients. Acitretin administered at 25 mg per day improved the patient’s blisters and reddening of the skin dramatically after six months [22]. In a different person, acitretin given at 25 mg per day caused moderate improvement of the skin lesion, but the improvement occurred rapidly [24]. Acitretin given at 25 mg per day helped a woman, who started showing signs of improvement after four weeks. The patient went on to achieve complete regression after three months [26]. However, in a group experiment, three patients were administered 25 mg of acitretin daily. The treatment moderately helped the lesions of only one patient, while it worsened the skin of the other two patients by exacerbating the lesions and the itchiness [23]. Different dosages of acitretin were experimented with as well. In one study in this review, acitretin was given at 10 mg per day, which led to the complete eradication of groin macerations and labial erosions [29]. In one case of an acitretin treatment, there was a slight improvement, but it was not linked to the acitretin, as the researchers thought it could have also simply been spontaneous improvement [34]. According to Table 1, in three case studies in this review, the use of acitretin had no effect on the Hailey-Hailey disease [35-37]. Acitretin has also been used in combination with other treatment methods. A case study involved the patient being prescribed 20-30 mg of acitretin per day, but then switched to 10 mg of acitretin in combination with cyclosporine of 1.2 mg/kg of body weight per day. The 20-30 mg dosage of acitretin caused the rash to become more painful, erythematous, and macerated. The 10 mg of acitretin in combination with cyclosporine dosage caused improvement in skin reddening and blisters in two days. After eight months, the Hailey-Hailey disease was relatively inactive [27]. Acitretin was started at 10 mg per day in a patient, then increased to 30 mg per day. The dosage of 10 mg caused the occasional occurrence of keratotic activity, but the 30-mg dosage of acitretin eradicated any occurrence of lesions or blisters [28]. Acitretin was administered in a case study at 25 mg per day, with narrowband phototherapy three times per week in flanks, axilla, under breasts, and mid and lower back. Narrowband phototherapy used UV radiation at 311-312 nm to reduce the growth of the damaged skin cells and to underlying inflammation [3]. After two months, the patient had significant improvement and almost complete clearance of affected skin in all areas. This result was maintained at five months follow-up [3]. In a different case study, acitretin was originally administered at 50 mg per day. The treatment method switched from 50 mg to 25 mg per day, with cyclosporine at 3.8 mg/kg of body weight per day. Four weeks after, the patient’s lesions showed more significant improvement than ever, and those in the axilla and groin disappeared completely. These results were sustained at six months follow-up [25]. While the use of acitretin has been shown to produce both desirable and undesirable effects in patients, the drug shows potential in becoming a more universal treatment method for Hailey-Hailey disease.

## Conclusions

Given all of the data from the studies compiled in this review, it is clear that surgical skin grafting demonstrates promise and potential to expand on our knowledge, and by extension treatment, of Hailey-Hailey disease. The value of the surgical skin graft is in the ability to remove the infected skin and replace it with healthy skin from a different area of the body. Additionally, the use of acitretin and tacrolimus can also prove to be valuable when looking for treatments of Hailey-Hailey. Tacrolimus can be used to treat reddening and itchy skin, which is a common effect of Hailey-Hailey disease. Acitretin is an immunosuppressant that combats inflammation and redness of the skin, actively improving the condition of the disease. There is still work to be done, specifically in tacrolimus and acitretin, in narrowing down best dosages and other compounds to be administered alongside them. Surgical skin grafting, coupled with tacrolimus and acitretin, could serve to be the root of prospective treatments for Hailey-Hailey disease. Further studies should be conducted with tacrolimus, acitretin, and surgical skin grafting to understand their long-term effects on mitigating the skin issues associated with Hailey-Hailey disease. These studies should be given more priority by dermatologists and healthcare leaders as a viable and practical treatment for Hailey-Hailey disease, and other disorders closely related to it.
